# Overcoming Addictions, a Web-Based Application, and SMART Recovery, an Online and In-Person Mutual Help Group for Problem Drinkers, Part 1: Three-Month Outcomes of a Randomized Controlled Trial

**DOI:** 10.2196/jmir.2565

**Published:** 2013-07-11

**Authors:** Reid K Hester, Kathryn L Lenberg, William Campbell, Harold D Delaney

**Affiliations:** ^1^Behavior Therapy Associates, LLCResearch DivisionAlbuquerque, NMUnited States; ^2^Presbyterian Medical GroupIntegrated Behavioral HealthAlbuquerque, NMUnited States; ^3^Psychology DepartmentThe University of New MexicoAlbuquerque, NMUnited States

**Keywords:** addictions, cognitive-behavioral program, Web application, SMART Recovery, mutual self-help groups

## Abstract

**Background:**

Overcoming Addictions (OA) is an abstinence-oriented, cognitive behavioral, Web application based on the program of SMART Recovery. SMART Recovery is an organization that has adapted empirically supported treatment strategies for use in a mutual help framework with in-person meetings, online meetings, a forum, and other resources.

**Objective:**

To evaluate the effectiveness of OA and SMART Recovery (SR) with problem drinkers who were new to SMART Recovery. Our experimental hypotheses were: (1) all groups will reduce their drinking and alcohol/drug-related consequences at follow-up compared to their baseline levels, (2) the OA condition will reduce their drinking and alcohol/drug-related consequences more than the control group (SR), and (3) the OA+SR condition will reduce their drinking and alcohol/drug-related consequences more than the control group (SR only).

**Methods:**

We recruited 189 heavy problem drinkers primarily through SMART Recovery’s website and in-person meetings throughout the United States. We randomly assigned participants to (1) OA alone, (2) OA+attend SMART Recovery (SR) meetings (OA+SR), or (3) attend SR only. Baseline and follow-ups were conducted via GoToMeeting sessions with a Research Assistant (RA) and the study participant. We interviewed significant others to corroborate the participant’s self-report. Primary outcome measures included percent days abstinent (PDA), mean drinks per drinking day (DDD), and alcohol/drug-related consequences.

**Results:**

The intent-to-treat analysis of the 3-month outcomes supported the first hypothesis but not the others. Participants in all groups significantly increased their percent days abstinent from 44% to 72% (*P*<.001), decreased their mean drinks per drinking day from 8.0 to 4.6 (*P*<.001), and decreased their alcohol/drug-related problems (*P*<.001). Actual use relationships were found for the OA groups, between SR online meetings and improvement in PDA (*r*=.261, *P*=.033). In addition in the OA groups, the number of total sessions of support (including SR & other meetings, counselor visits) was significantly related to PDA (*r*=.306, *P*=012) and amount of improvement in alcohol-related problems (*r*=.305, *P*=.012). In the SR only group, the number of face-to-face meetings was significantly related to all three dependent variables, and predicted increased PDA (*r*=.358*, P*=*.*003), fewer mean DDD (*r*=*-*.250*, P*=.039), and fewer alcohol-related problems (*r*=-.244*, P*=.045), as well as to the amount of improvement in all three of these variables. Six-month follow-ups have been completed, and the results are currently being analyzed.

**Conclusions:**

These results support our first experimental hypothesis but not the second or third. All groups significantly increased their PDA and decreased both their mean DDD and their alcohol-related problems, which indicates that both interventions being investigated were equally effective in helping people recover from their problem drinking.

**Trial Registration:**

Clinicaltrials.gov NCT01389297; http://clinicaltrials.gov/ct2/show/NCT01389297 (Archived by WebCite at http://www.webcitation.org/6Hh5JC7Yw).

## Introduction

### Online Interventions for People With Alcohol and Drug Problems

In the past decade, there has been a marked rise in the number of online resources available to individuals with alcohol and drug problems, and evidence has steadily mounted to support their use [1-3]. One frequently recognized benefit of this trend is that individuals who might not otherwise seek treatment will consider an online intervention [4]. The Internet also makes interventions available to drinkers who—whether due to physical infirmity, geographic isolation, or lack of resources—might have difficulty accessing traditional treatment services. As online interventions have become more prevalent, people have used these interventions on a scale that would overwhelm conventional resources [5].

Online interventions are used in a variety of contexts, from clinical settings to college dorms to free access on the Internet. They may be presented as stand-alone treatments, as the first step in a stepped model of care, as an adjunct to traditional care, or as a hybrid [2,5,6]. The form and content of these Web-based interventions vary widely, from simple text-based adaptations of brief screening instruments that take a minute or two to complete, to multisession, multimedia, interactive interventions that extend over several hour-long sessions [7-9].

### Alternative Protocols

While the predominant paradigm for conceptualizing addictive behaviors in the United States is the 12-step model (eg, Alcoholics Anonymous, Narcotics Anonymous, etc), a significant proportion of individuals who are looking for help with their addictions reject 12-step programs for a variety of reasons [10]. At least some of these individuals are interested in viable alternative recovery options, often preferring approaches that provide them with more flexibility in how they define and address their addictive behavior(s). SMART Recovery (Self-Management And Recovery Training) [11] provides such individuals with a protocol that, like a 12-step program, employs the use of an interactive group component (either in person or through the use of Web-based chat rooms and a forum) while using the framework of the 4-point program (described below). However, SMART Recovery fundamentally differs from the 12-step model in that (1) “participants learn tools for addiction recovery based on the latest scientific research”, (2) it avoids labeling (eg, “alcoholic” or “addict” unless individuals themselves accept that label), and (3) it does not conceptualize addiction as a disease per se (but is accepting of members’ views of addiction as a disease) [12]. Anecdotal evidence from SMART Recovery meetings indicates that these aspects of the program draw participants to SMART Recovery (A.T. Horvath, personal communication, 12/2/08).

### The Overcoming Addictions Web Application

The Overcoming Addictions Web Application (OA) is an abstinence-focused, cognitive-behavioral Web application [13] that we developed for SMART Recovery [11] that is based on its protocol. The program has parallel but separate modules for alcohol, marijuana, opioids, stimulants, and compulsive gambling. The interactive exercises in OA include tasks that focus on the 4-point program of SMART Recovery as well as additional activities to enhance motivation for change; track urges over time (with feedback); practice mindfulness exercises for preventing relapse [14], set goals, and make Change Plans [15]. Most other online interventions are brief interventions designed to increase users’ motivation for change. OA is unusual in the realm of online interventions in that it focuses on the action stage of change.

To evaluate the effectiveness of OA and SMART Recovery, we conducted a randomized clinical trial (trial registration NCT01389297). Our experimental hypotheses were that (1) all groups will reduce their drinking and alcohol/drug-related consequences at follow-up compared to their baseline levels, (2) the OA condition will reduce their drinking and alcohol/drug-related consequences more than the control group (SR), and (3) the OA+SR condition will reduce their drinking and alcohol/drug-related consequences more than the control group (SR only).

## Methods

### Description of the Intervention: SMART Recovery

SMART Recovery’s protocol for change combines motivational enhancement with cognitive-behavioral principles and strategies for behavior change. Its 4-point program focuses on (1) building and maintaining motivation, (2) dealing with urges, (3) managing thoughts, feelings, and behaviors, and (4) cultivating a lifestyle balance (of short- and long-term rewards) to prevent relapse.

SMART Recovery’s program uses a common set of strategies to address all addictive behaviors. Their rationale for this is based on two aspects of addiction: (1) common etiological factors in both the development and maintenance of addictive behaviors (eg, affect regulation) [16], and (2) the broad applicability of cognitive-behavioral and motivational strategies that are supported by outcome research across addiction treatments [17]. For instance, alcohol, drugs, and compulsive behaviors like gambling produce powerfully reinforcing changes in affective states, at least on a short-term basis [18]. Identifying these immediate positive consequences is an important step in developing more adaptive alternatives.

SMART Recovery’s menu of cognitive-behavioral and motivational strategies has been adapted from treatment interventions and it “evolves as scientific knowledge in addiction recovery evolves” [11]. Its elements are designed to help members address issues ranging from basic motivation for change to qualitative lifestyle changes intended to reduce the appeal of, and engagement in, harmful addictive behaviors.

SMART Recovery has a large and active online presence. In 2012, their website had, on average, 69,786 visits per month and 991 new subscribers on their online forum each month. The message boards now have over 50,000 registered users (a 130% increase in the last 2 years) (S Alwood, personal communication, 1/22/13). In addition to their online presence, they have over 800 in-person support groups worldwide [19].

### Description of the Intervention: Overcoming Addictions

OA is an action stage program designed to help users learn how to achieve and maintain abstinence. It is a self-directed and interactive Web application developed to be used either as a stand-alone intervention, an adjunct to attending SMART Recovery meetings, or as an adjunct to professional therapy for addictions (see Multimedia Appendix 1). Participants could access OA anywhere or anytime they had an Internet connection. Reviewers wishing to access the program can contact the senior author for a reviewer’s access login.

**Figure 1:**
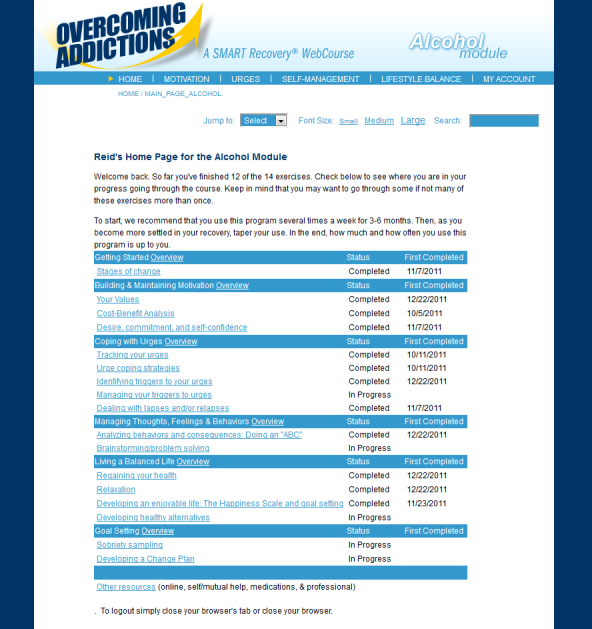
Overcoming Addictions Web app home page.

The OA program contains and extends the elements of the 4-point program of SMART Recovery. Prior to registering, a user can read an overview of the program and its relationship to SMART Recovery. During registration, users provide a first name, gender, email address which is also their login username, and password. Once registration is completed, the program creates a new record in its database and personalizes content for that user (eg, Welcome back, John). The user is then taken to a homepage that lists all of the program’s exercises and materials that are grouped by focus. The user can access any module of the program in any order that he or she chooses (see Figure 1 for a screenshot of a user’s home page).

The first module, *Getting Started* gives an overview of the program, provides a discussion of the Stages of Change [20], and suggests exercises based on the individual’s perceived stage.

The second module, *Building and Maintaining Motivation for Change*, contains a values exercise, a decisional balance exercise that asks users to weigh the pros and cons of changing, and a cost-benefit analysis exercise that is designed to elicit “change talk” from the user (see Multimedia Appendixes 2-5).

The third module, *Dealing with Urges and Cravings*, begins with a brief discussion of urges and their relationship to sobriety and lapses/relapses. It teaches users to self-monitor their urges to use, noting the date, time, intensity, and duration of the urge, the trigger to the urge, how they handled the urge, and their reactions to how they handled it. Users are able to print out a page of self-monitoring cards so that they can easily collect these data as urges happen during their day. Later, when users enter their self-monitoring data, they are provided with graphic feedback about the frequency, intensity, and duration of their urges over time. This feedback can help users see whether they’re making progress in experiencing fewer urges over time. If a user is not experiencing a gradual decline in the frequency, intensity, or duration of urges over time, the program suggests they consider additional or alternative urge-coping strategies. The module also contains the urge-coping strategies recommended by SMART Recovery, empirically supported mindfulness/relaxation exercises, and a section on medications that can help reduce urges and cravings.

In addition, exercises are available to help users identify and manage the triggers that precede urges. Identifying triggers is similar to the first step in a functional analysis of drinking behaviors [21], and users are encouraged to develop plans for managing the triggers they personally identify. It is a complex module because triggers range from simple (eg, wanting to drink more with some friends than others) to complex (eg, negative mood coupled with poor coping skills). For each domain of triggers, the program presents strategies that others have found to be helpful.

The fourth module is *Self-Managing Thoughts, Behaviors, and Feelings*. There are three exercises in this module: (1) the “ABCs” of Rational Emotive Behavior Therapy (REBT) [22], (2) unconditional self-acceptance, and (3) problem solving. The ABCs of REBT section has multiple subcomponents: dysfunctional beliefs, coping statements, changing one’s self-talk to change one’s feelings, and the process of analyzing and correcting dysfunctional beliefs that produce negative affect [23] (see Multimedia Appendix 6).

The fifth module is *Lifestyle Balance for Preventing Relapse*. This module has five components: regaining one’s health, relaxation, goal setting, social and recreational activities, and other relapse prevention strategies. The section on regaining one’s health focuses on eating and sleeping well, and exercising. The section on relaxation training targets both those with high levels of trait anxiety as well as those sensitive to situation specific anxiety (eg, when experiencing urges to drink/use). The goal-setting component focuses on setting short-term goals that are specific, measurable, achievable, realistic, and timed (eg, once a day). The section on social and recreational activities helps individuals consider and sample enjoyable and rewarding prosocial activities that are compatible with their goals and values and that make a sober life more rewarding than drinking, using drugs, or engaging in other addictive behaviors. The section on relapse prevention strategies presents relapse as a learning experience (eg, the Abstinence Violation Effect [24]) and offers some additional strategies that have not been covered in the other modules.

The appearance of the site is pleasant and uncluttered. Content is delivered via text, embedded videos and audio files, links to other sites, pop-up windows, and graphic feedback charts. The site is structured in the hybrid style, meaning that all content is available from a central matrix homepage. Once users choose a content area, their exploration of the content is constrained by tunnels that direct them through the various exercises. At the conclusion of an exercise, users have the option of continuing to the next recommended activity, or they may return to the homepage.

Like most computer-delivered interventions, users are free to access as much program content, in any order, and whenever they choose. Their use is supported by a customizable SMS (short message service) text messaging and email system that prompts them to log onto the program, reminds them of their plans for managing triggers, reiterates their reasons for staying sober, or presents motivational thoughts. These personalized messages can be delivered daily at user-defined times.

### Experimental Design

#### Recruitment

Participants were recruited through a home page announcement on SMART Recovery’s website, announcements of the study at SMART Recovery face-to-face and online group meetings nationally, and on their blog. We also placed a thread on the SMART Recovery online forum announcing the study and invited individuals who were new to SMART Recovery to participate in the study.

#### Inclusion Criteria

Criteria were (1) a minimum age of 18, (2) drinking 5 or more, or 4 or more for women, standard drinks on at least one occasion in the last 90 days, (3) have an Alcohol User Disorders Identification Test (AUDIT, [25]) score of 8 or higher, (4) new to Smart Recovery (ie, are just joining or have joined within the last 4 weeks), (5) have a computer at home with Internet access, and (6) have a primary treatment goal to abstain from drinking.

#### Exclusion Criteria

Criteria were (1) court-mandated DWI offenders, (2) a current diagnosis of drug dependence or consider themselves to be drug dependent, (3) a reported diagnostic history of psychosis or bipolar disorder not medically managed, (4) exhibit evidence of significant cognitive impairment from brain dysfunction (based on self-report and research assistant’s clinical judgment during screening), (5) have an English reading level below the 8^th^ grade, (6) are unwilling or unable to be available for follow-up appointments at 3 and 6 months from enrollment into the study, and (7) unwilling or unable to provide one Significant Other (SO) for corroboration of participant’s self-reported drinking and drug use (if any).

A minimum AUDIT score of 8 suggests that the person is at least “at risk” for alcohol-related problems. It is important to recruit participants who are new to SMART Recovery to evaluate its *initial* effect on their drinking, drug use, and related consequences. A computer with Internet access at home is necessary for participants to use the Web application.

Regarding exclusion criteria, court-mandated DWI offenders are often required to attend self-help groups, and we were concerned that these treatment-mandated offenders would have no motivation to continue beyond their mandated participation. Furthermore, such a group could prove to be difficult to find at follow-up assessments. Since the primary focus is on drinking, those with either a current diagnosis of drug dependence *or those who consider themselves to be drug dependent* were excluded. Criteria 3 and 4 reflect the need for study participants who can reason, recall, and comprehend information both in the experimental and control group. The reading level of the OA Web application is set at an 8^th^ grade level. Potential participants were asked about their educational level to ensure they would understand the material presented. Last, we contacted participants’ SOs both to corroborate their self-report of their drinking and drug use as well as to provide them with resources that may be helpful to them in supporting their loved one’s changes.

#### Screening

Potential participants were screened over the phone using a questionnaire addressing the inclusion criteria 1 and 4-6 and exclusion criteria questions 1-7. The research assistant administered the AUDIT over the phone and asked two quantity/frequency questions, “How often have you had 5 or more (4 or more for women) standard drinks (explained briefly) in the last 90 days?” and “During the last 90 days, have you drank as often as once a month?” A response of one or more times to both questions was sufficient to be included in the study. These two screening questions were adapted from those used by Cherpitel [26], who found them sensitive and specific in screening for alcohol abuse and dependence. We also included a question regarding suicidal thoughts, intent, or behaviors. If a participant endorsed this item, we discussed ways to access support (eg, National Suicide Hotline).

We emailed potential participants a demographic form, a patient locator form, a copy of the Brief Symptom Inventory (BSI) [27], and an Informed Consent form. BSI scores were reviewed prior to enrolling potential participants in the study; if their scores were elevated and the participant reported significant levels of distress, they were encouraged to access professional support [28]. Potential participants who screened positive, had a consenting SO, and signed the Informed Consent form were randomized to either the experimental or the control groups. The timeline for the post-baseline assessments began when the participant completed his or her baseline interview.

#### Randomization

We used a computer-generated stratification process for randomization. Participants were classified into blocks based on gender and ethnicity (white, hispanic, or other). Within each block, participants were randomly assigned to one of the three groups. After the first 3 months, we stopped randomizing participants to the OA only group, and we started encouraging those who had been assigned to this group to attend SR meetings. We did this because recruitment was slow and feedback from referral sources at SMART Recovery indicated that many potential participants were unwilling to be randomized to a condition that asked them to not attend SR meetings.

#### Assessments

We used the Timeline Follow-Back (TLFB) [29,30] to measure quantity/frequency of alcohol, drug, and tobacco use. The 90-day TLFB was administered at baseline and again at 3- and 6-month post-baseline, which provided continuous data for a total of 9 months. The TLFB was also used to collect data on study participants’ attendance at SMART Recovery meetings and other recovery oriented activities in which they may have engaged. We used the Inventory of Drug Use Consequences (InDUC) to measure both lifetime and recent (last 3 months) alcohol- and drug-related consequences. The psychometric properties are described in the manual for the Drinker’s Inventory of Consequences (DrInC) that was developed for Project MATCH [31]. The InDUC includes 5 subscales measuring interpersonal, intrapersonal, and physical consequences, impulse control, and social responsibility.

#### Baseline Interview

After participants completed and returned the consent form, BSI, Participant Locator, and demographics forms, they were scheduled for a baseline interview. We used the GoToMeeting website to complete the interview. This program allows sharing of the interviewer’s screen so the assessment can be viewed by both parties. Participants used the TLFB calendar generated to prompt recall of their prior 3 months of drinking as the RA entered their data in a Web application that we developed for collecting data for this study, the Drinker’s Evaluation. Participants then were guided to the InDUC and asked to complete it. At the completion of the interview, they were randomly assigned to a group. Participants and research staff were not blinded to group allocation.

Participants often wanted to discuss their histories and current struggles during the assessments. In order to limit the effect of the assessment interaction, RAs responded empathically but as briefly as possible, without soliciting further processing by the participant. Further, RAs directed, as indicated, that the participant seek help from the interventions being tested in the trial. All participants received a welcome email to the study. For those assigned to the OA conditions, there was a link to the OA registration page. For those assigned to meetings, a link to the SMART Recovery website was provided to facilitate locating available meetings.

#### Treatment Exposure and Fidelity

Treatment fidelity in the Web application is maintained by the nature of the technology used. All participants in the group who used the OA Web application were exposed to the same program. However, because participants were able to use the OA program and any module in it as often as they chose, the amount of exposure to the intervention, the number of modules used, and the way in which modules were used varied from participant to participant. Further, there was no a priori minimum number of sessions or modules a participant must have completed to be considered to have received the intervention. Further analysis of participants’ engagement with the intervention and correlations with treatment outcome will be reported in Part 2, which will include 6-month outcomes.

Fidelity in the SR meetings and online resources also varied in two ways. First, the SMART Recovery website underwent substantial improvements in content and navigation during the course of the trial and the availability of face-to-face and online meetings increased. Second, just as with the OA app, participants decided how much or how little to avail themselves of these resources.

#### Maximizing Compliance With Protocols

The OA program has an integrated email feature that contacts users who have not logged into the program in a week. A personalized email encourages participants to log in and resume their progress through the program. There was no protocol for encouraging participants to attend their SMART Recovery meetings.

This study was approved by the Presbyterian Health care Services Institutional Review Board. Consent was obtained by emailing consent forms and asking for participant signature and witness signature. The consent outlined the nature and extent of participation in the trial. Participants were reminded their participation was voluntary, and they could withdraw from the study at any time. In addition, participants were told they would not be identified to anyone outside of the study staff at any time for any reason. Participants returned the consent forms via mail or scanned the documents in and emailed them.

### Statistical Methods

Consistent with intent-to-treat analyses, we examined the entire sample as well as examined changes within the randomly assigned groups, both with and without imputation to account for missing data. In addition, we formed groups based on their use of either SMART Recovery meetings or the OA application to examine actual use outcomes.

## Results

**Figure 2:**
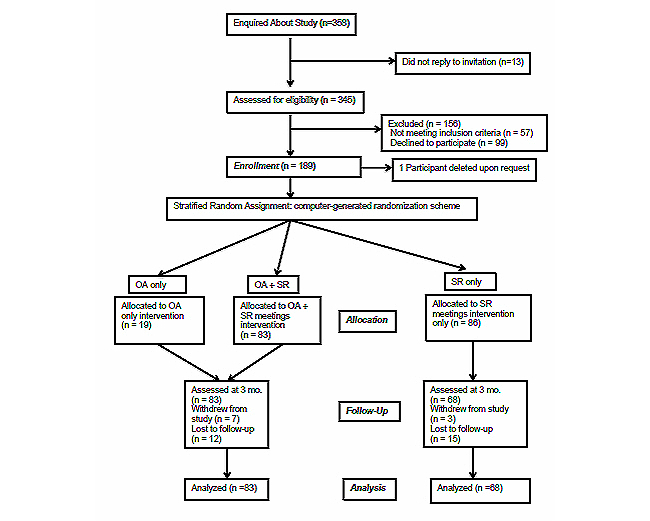
Flowchart of participant flow and follow-up.

Figure 2 illustrates the flow of participants through the study. Approximately 358 people new to SMART Recovery contacted us and expressed interest in participating in the study. Of these, 345 participants completed an initial screen with research staff. Of these, 99 were not interested, 19 did not meet the inclusion criteria, and 38 were excluded. The initial screening forms were emailed to potential participants and returned either via fax or scanned and emailed. In total, 195 participants completed the initial consent process, submitted their completed forms, and were scheduled for an initial assessment. Of these, 189 completed the initial assessment and were randomly assigned to one of three groups. One participant requested all data be removed from the study 24 hours after completing the initial interview, and we granted the request. Nineteen participants were assigned to the OA only condition, 83 were assigned to the OA plus SMART Meetings condition (OA+SR), and 87 were assigned to the SMART Meeting only condition (SR) for a total n=189.

Recruitment began September 12, 2011 (3 pilot participants were recruited in the first 2 weeks of the study), and ended August 1, 2012. Three-month follow-ups were completed November 1, 2012. Six-month follow-ups were completed March 14, 2013.

Table 1 presents the general characteristics of the participants as a whole and by group assignment. There are several striking aspects of this group of participants. First, 60.6% (114) were female. Second, the mean education level was 16 years (SD 2.4) indicating this population generally had a college education. Third, the mean AUDIT score of 24.7 (SD 8.1) is in the high range and indicates that this group would be recommended for a more extensive diagnostic evaluation for alcohol dependence. In addition, the mean score for the BSI for men was 15.62 (SD 11.4) and for women was 18.54 (SD 13.7) suggesting that many of the participants were experiencing psychological distress when they completed the initial interview. There were no significant differences between groups on any variable.

Of the 189 participants who completed random assignment and baseline interviews, 151 (83%) completed the 3-month interview. Of the 37 for whom we do not have 3-month follow-up data, 10 withdrew from the study, and 27 were lost to follow-up. Of the 151 with 3-month follow-up data, 83 were assigned to the OA and OA+SR groups, and 68 were assigned to the SR group.

**Table 1:** Pretreatment characteristics of participants by group.

Variable		Overall	Group		
			SR, n=86	SR+OA, n=83	OA, n=19
Female, n (%)		114 (60.6)	52 (61)	50 (60)	12 (63)
Age, M (SD)		44.3 (10.9)	43.4 (10.6)	44.6 (11.1)	48.3 (8.4)
**Ethnicity, n (%)**					
	White	170 (90.4)	76 (88.4)	77 (92.8)	17 (89.5)
	Hispanic	5 (2.7)	3 (3.5)	1 (1.2)	1 (5.3)
	Other	7 (6.9)	7 (8.1)	5 (6.0)	1 (5.3)
Education, M (SD)		16.1 (2.4)	15.93 (2.5)	16.0 (2.3)	17.3 (2.1)
AUDIT^a^, M (SD)		24.7 (8.1)	24.8 (8.1)	23.95 (8.2)	27.4 (7.2)
BSI^b^, M (SD)		17.4 (12.9)	19.35 (12.5)	15.95 (13.6)	14.8 (11.0)
InDUC^c^, M (SD)		41.4 (17.9)	42.2 (19.0)	40.6 (17.5)	40.8 (15.6)

^a^Alcohol User Disorders Identification Test.

^b^Brief Symptom Inventory.

^c^Inventory of Drug Use Consequences.

### Lost to Follow-Up

We compared baseline characteristics between those completing the 3-month follow-up and those who were lost to follow-up. No differences existed between those followed up and those lost to follow-up on the following continuous variables at baseline: age, mean drinks per drinking day, AUDIT, BSI total, InDUC recent score, or PDA. No differences across groups existed on the categorical variables of group assignment, gender, or ethnicity. Only education level demonstrated a significant difference, with those who were contacted at 3 months reporting having completing more years of education (16.3) than those lost to follow-up (15.3), *t*_186_=2.20, *P*=.029.

### Intent-to-Treat Analysis

Separate repeated measures analyses of variance were conducted to assess for significance of the change over time. Our three outcome measures were Percent Days Abstinent (PDA), Mean Standard Drinks per Drinking Day (DDD), and the InDUC Recent Total score (InDUC). Improvement over all groups from baseline to 3 months was highly significant on all three dependent variables: PDA, *F*_1,149_=160.93, *P*<.001, with the mean PDA increasing from 44% to 72%; DDD, *F*_1,149_=61.73, *P*<.001, with the mean decreasing from 8.0 to 4.6; and InDUC, *F*_1,149_=122.28, *P*<.001, with the overall mean decreasing from 40.8 to 19.5. However, none of the tests of group differences in change over time approached significance, *F*≤1.0. The within-group effect sizes (Cohen’s *d*) are presented in Table 2. Tests of effects of treatment group were carried out both as tests of Group x Time in a repeated measures approach and as ANCOVAs. None of the tests that would have been indicative of differential treatment effects approached significance.

In addition to these primary analyses conducted on participants having follow-up data, data were reanalyzed after values were imputed for participants having missing data using predictive mean matching [32], and results were essentially unchanged. That is, tests of time were again highly significant, and tests of treatment x time did not approach significance.

### Actual Use Analysis

Because study participants could use these resources as much or as little as they chose to, we examined changes over time and treatment group effects for those actually using the resources of the assigned treatment, and examined relationships between engagement (eg, logging into OA, attending SR and other meetings, and counselor visits) and outcomes.

#### Time and Treatment Group Effects for Those Actually Treated

Although it was unclear what criterion to use to consider a participant treated, 59 (71%) of the 83 OA+SR participants completing the 3-month follow-up had completed 2 or more OA sessions, and 58 (85%) of the 68 SR participants completing the 3-month follow-up had attended 2 or more SR meetings. Using these definitions of being actually treated, improvement of treated participants over all groups from baseline to 3 months was highly significant on all three dependent variables: PDA, *F*_1,115_=139.71, *P*<.001, with the mean PDA increasing from 44% to 73%; DDD, *F*_1,115_=55.04, *P*<.001, with the mean decreasing from 8.3 to 4.4; and InDUC, *F*_1,115_= 93.95, *P*<.001, with the overall mean decreasing from 39.6 to 18.7. However, none of the tests of group differences in change over time approached significance, *P*>.10.

**Table 2:** Means and within group effect sizes for each outcome variable.

Variable & Group		Baseline	3-month follow-up	Improvement	Within group effect size *d* ^a^
**Percent days abstinent (PDA)**					
	OA+SR	43.83	73.32	29.49	1.00
	SR only	43.61	71.18	27.57	.91
**Std. drinks per drinking day (DDD)** ^b^					
	OA+SR	7.88	4.59	3.29	.77
	SR only	8.25	4.66	3.59	.78
**InDUC recent score** ^c^					
	OA+SR	40.19	19.96	20.23	1.13
	SR only	41.47	18.88	22.59	1.19

^a^Cohen’s *d.*

^b^Standard drink is equal to 12 oz (355 mL) of 5% beer, 5 oz (149 mL) of 12% wine, or 1.5 oz (44 mL) of 80 proof liquor.

^c^Alcohol-related problems.

#### Comparisons of Those Using Only OA With Other Groups

**Figure 3:**
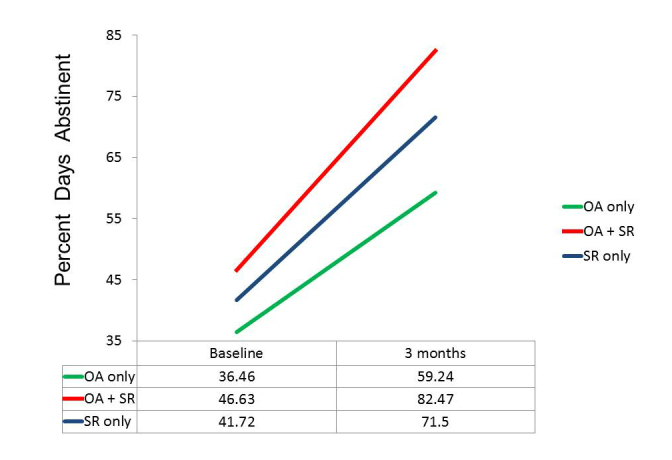
Actual use groups: Percent days abstinent.

**Figure 4:**
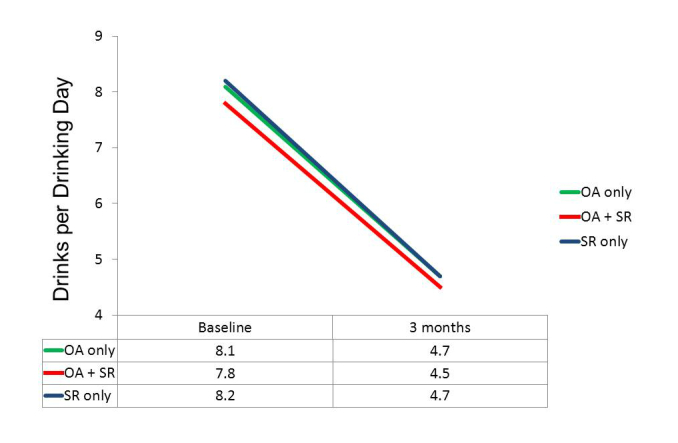
Actual use groups: Mean standard drinks per drinking day.

**Figure 5:**
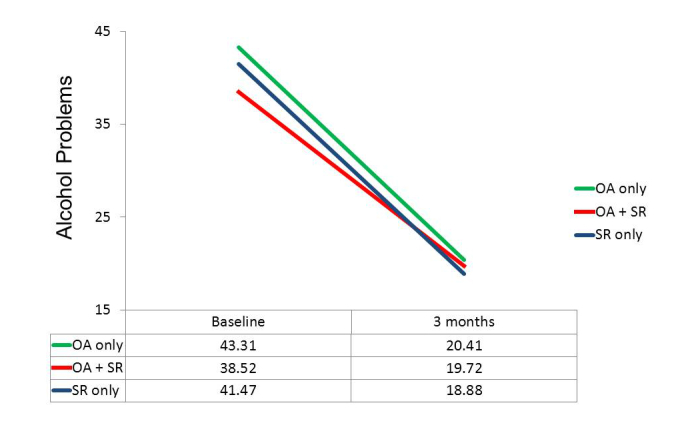
Actual use groups: Alcohol-related problems.

Although we had to abandon our initial design, which included a group that would have used only OA without having the option of participating in any SR meetings, there were 29 of the 83 participants in the OA conditions who did not take part in SR meetings. This allowed post hoc comparisons to be made among three groups: those using only the OA app (n=29), those who both used the OA app and attended SR meetings (n=54), and those randomly assigned to SR only. These three groups did not differ significantly in composition by gender, ethnicity, age, or education. Although there were no significant differences in mean baseline values on our three primary dependent variables, the trend in each case was for those in the OA only group to be more impaired initially than those who attended SR meetings. Repeated measures ANOVAs again indicated highly significant changes over time on all three dependent variables (*P*<.001), but, more importantly, tests of the group x time interaction were nonsignificant. As suggested by the plots of means in Figures 3-5, the test for differential change across the three groups did not approach significance for DDD, *F*_1,141_=0.09, *P*=.919, or for InDUC, *F*_1,141_=0.34, *P*=.713. For PDA, while the omnibus test of the group x time interaction was nonsignificant, *F*_1,141_=2.04, *P*=.134, the plot of means revealed more separation of the groups. In fact, the main effect of groups on PDA was significant, *F*_2,141_=3.10, *P*=.048, because the overall mean PDA in the OA+SR group (63.4) was greater than the average of the other two groups (53.5), *F*_1,141_=4.65, *P*=.033. However, this resulted in part from the higher mean PDA at baseline in the OA+SR group, because there was not significant evidence of differential improvement across groups. That is, tests of interaction contrasts indicated that not only was the improvement in the SR only group (27.6) not different from that in the OA only group (23.2), *F*_1,141_=0.51, *P*=.475, but the improvement in the OA+SR group (32.9) was also not significantly larger than the average improvement of the other two groups (25.4), *F*_1,141_=2.41, *P*=.122.

#### SR Meetings or Other Support

Was the number of SR meetings, other meetings, and counselor visits predictive of the 3-month outcomes or of the improvement from baseline to 3 months for participants in the two groups? There was evidence of this, with the evidence being stronger in the SR only group than in the OA+SR condition.

Although the trend was for the SR only group to have more days of face-to-face meetings (3.31), more days of SR online meetings (5.90), and more days of Any Support (14.85) than the combined OA group (1.82, 4.42, and 12.80, respectively), these were not significantly different across conditions. For the SR only condition, the number of days of face-to-face meetings reported at 3 months was significantly predictive of all 6 of these outcome measures: PDA at 3 months (*r*=.358, *P*=.003), mean DDD (*r*=-.250, *P*=.039), and InDUC Recent Total at 3 months (*r*=-.244, *P*=.045), as well as improvement in PDA (*r*=.274, *P*=.024), mean DDD (*r*=.478, *P*<.001), and improvement in InDUC Recent Total (*r*=.403, *P*=.001). On the other hand, for this group, number of days of SR online meetings was *positively* related to mean DDD at 3 months (*r*=.260, *P*=.032). Number of days of any support for the SR group was positively related to PDA at 3 months (*r*=.260, *P*=.032) as well as to improvement in PDA (*r*=.304, *P*=.012).

In the OA+SR group (ie, excluding the 16 participants assigned to the OA only condition), neither days of face-to-face meetings nor days of SR online meetings were significantly related to any of the outcomes at 3 months or to improvement in those variables from baseline. The variable most predictive of outcomes for this group was the number of days of any support, which was significantly related to PDA at 3 months (*r*=.306, *P*=.012) and to improvement in InDUC Recent Total (*r*=.305, *P*=.012). In addition, number of days of SR online meetings was predictive of improvement in PDA (*r*=.261, *P*=.033). Relevant to the anomalous finding of the *positive* correlation in the SR group between SR online meetings and mean DDD, the correlation between these variables in the OA+SR group was slightly negative using all 67 subjects (*r*=-.055). However, if the one subject in this group who reported 83 days of online SR meetings were excluded, the correlation between number of SR meeting days and mean DDD would have been positive in this group as well (*r*=.112, *P*=.372).

#### Number of OA Sessions

The OA sessions completed variable was available only for those participants in the OA conditions. Participants logged into the OA program, on average, 7.2 times (SD 6.4). To assess whether there was evidence for an engagement-response relationship the number of sessions completed in the first 90 days was correlated with the values of the primary outcome variables at 3 months and with the improvement in those variables from baseline to 3 months. As shown in Table 3 below, none of these six correlations was significant. Number of days of SR online meetings was significantly predictive of improvement in PDA for the OA participants (*P*=.025). Furthermore, number of days of any support was significantly correlated with PDA at 3 months (*P*=.006), and with improvement in InDUC Recent Total (*P*=.045).

#### Corroboration of Self-Report Drinking by Significant Others

We collected data from 147 significant others (SO) for baseline and 3-month follow-up. In short, the reports of the SOs mirrored those given by participants. Examining the effects of time and treatment, similar to the participants, the SO data demonstrated a highly significant effect of time and nonsignificance for treatment x time effect. For mean DDD, the test of time yielded *F*_1,145_=105.25, *P*<.001, whereas the time x treatment interaction was nonsignificant, *F*_1,145_=0.32, *P*=.573. For PDA, the test of time yielded *F*_1,145_=140.45, *P*<.001, whereas the time x treatment interaction was nonsignificant, *F*_1,145_=1.42, *P*=.236.

Correlations between participants’ self-report and SOs’ reports were consistently highly significant: mean DDD at baseline, *r*=.651, *P*<.001; PDA at baseline, *r*=.654, *P*<.001; mean DDD at 3 months, *r*=.662, *P*<.001; PDA at 3 months, *r*=.519, *P*<.001.

**Table 3:** Correlations between various resources available and outcome variables at 3 months, significant relationships noted.

Variable	PDA	DDD	InDUC	PDA Improvement	DDD Improvement	InDUC Improvement
# OA sessions baseline to 90 days	-.003	-.041	.067	-.074	.073	-.212
# SR face-to-face meetings	.138	.067	-.023	.146	.020	.074
# SR online meetings	.185	-.055	-.170	.246^a^	.079	.151
# of counselor visits	.201	-.098	.025	-.070	.031	-.019
# of other meetings	.140	-.099	-.066	.035	-.037	.170
Total of any support	.298^a^	-.115	-.148	.187	.036	.220^b^

^a^ *P*<.01.

^b^ *P*<.05.

## Discussion

### Principal Results

The experimental hypotheses were that (1) all groups will reduce their drinking and alcohol/drug-related consequences at follow-up compared to their baseline levels, (2) the OA condition will reduce their drinking and alcohol/drug-related consequences more than the control group (SR), and (3) the OA+SR condition will reduce their drinking and alcohol/drug-related consequences more than the control group (SR only). These results support our first experimental hypothesis but not the second or third.

All participants in this randomized clinical trial improved on outcomes that are important to recovery from problem drinking. Participants significantly increased their percent days abstinent per week, significantly reduced the number of drinks they consumed on the days when they did drink, and the number of alcohol-related problems. The mean effect sizes of reductions in drinking and alcohol-related problems, averaging across the three dependent variables, were 0.97 for the OA+SR group and 0.96 for the SR only group, both being in the large range (0.8+). These statistically significant results are clinically significant. We also consider it remarkable that participants with this degree of heavy drinking made these changes over the period of 3 months.

The mean reduction in alcohol-related problems was more than 50%. While there are no norms yet for the InDUC, we have norms for the DrInC from our online Drinker’s Check-up [33,34]. The only difference between the two instruments is that the InDUC adds the words “or drugs” to the questions. Since the level of drug use in the participants in this study was low (only 25% reported any drug use at baseline and the frequency of drug use in the period had a mean of 0.3 instances and the maximum number of instances of use for any participant was 3 in 90 days), we can assume some comparability between the InDUC and DrInC scores. Assuming this comparability, participants went from the 82^nd^ percentile at baseline to the 50^th^ percentile at follow-up.

The correlations between attendance at SR meetings, other meetings, and counselor visits and outcomes are consistent with the perspective that the more assistance participants availed themselves of, the better their outcomes.

The analyses of how participants actually engaged with these resources present a similar picture. Significant improvements were seen on all outcome measures and no significant differences between those who only used the OA app, those who only attended meetings, and those who used both resources. The trend towards greater improvement in PDA in the group that used both resources (OA+SR) seems to be due in part to a higher level of abstinence at baseline. Conversely, the OA app only group had the lowest level of abstinence at baseline. This begs the question of whether there were other differences in this group that led them to not attend meetings. We can only speculate at this point that perhaps this group had a higher level of anxiety that may have led them to avoid attending meetings where the social norm is accountability and self-disclosure. We plan to examine this in subsequent analyses.

Attending SMART Recovery meetings appears to “work” as well as the Overcoming Addictions Web app (which is based on SMART Recovery). The reverse is also true. Having these two different ways to deliver the SMART Recovery protocol gives problem drinkers options with regards to how they learn to achieve and maintain abstinence. Some participants in our study preferred using the Web app alone. Others preferred to attend meetings. This is likely to be the case with people coming to the SMART Recovery website for the first time and considering their options. Having both protocols with equal effectiveness available increases the chances that individuals can find a path to recovery that suits them. It also increases opportunities for problem drinkers who may have limited geographical access to a face-to-face mutual support group and to those who are not inclined to attend group support meetings.

### Comparison With Prior Work

The lack of differences between assigned groups in either the intent-to-treat analyses or the actual use analyses was surprising from the traditional perspective that more intervention results in better outcomes. While this is often the case in addictions treatment outcome research, it is not always the case with freestanding online interventions. In our previous randomized clinical trial of Moderate Drinking with less dependent drinkers, we did not find a relationship between number of sessions logged in and outcomes [9,35].

On the other hand, Carroll and colleagues did find an additive benefit from their computer-delivered intervention, Computer-Based Training for Cognitive Behavioral Therapy (CBT4CBT) [36]. Their study population, however, was with individuals seeking treatment for substance dependence at a community clinic, which is a population different from individuals seeking assistance online who are not entering treatment for substance abuse.

The prevalence of women (60%) in this study is also consistent with our previous studies of Moderate Drinking (56%) and of our brief motivational intervention, the Drinker’s Check-up (48%) [37]. This is remarkable given the epidemiological data indicating that the ratio of problem drinkers by gender is 65% male and 35% female [38], although it does reflect findings that the prevalence of problem drinking among women is increasing [39].

### Limitations

There are a number of limitations to this study. First, we did not have a no-intervention control group. While we found it neither practically nor ethically feasible to include such a group in our study, the lack of such a comparison group prevents us from being assured that the treatment assigned was the cause of the improvement. Second, we could not separate out the effects of assessment reactivity that, based on participants’ anecdotal reports, did sometimes occur as a function of the baseline evaluation. Third, study participants had, on average, a high level of education (mean 16 years). While this seems to be consistent with the heavy drinkers who affiliate with SMART Recovery, it potentially limits the generalizability of the outcomes in populations with lower levels of education. Fourth, the requirement for an SO to corroborate the participant’s self-report of drinking may have further limited the sample. We considered that requirement necessary though as we had no other way to confirm participants’ self-reports of their drinking.

### Conclusions

Both the Overcoming Addictions Web application and the use of the meetings and other resources of SMART Recovery are effective in helping people recover from heavy problem drinking.

## Multimedia Appendix 1

Overcoming Addictions overview video.

## Multimedia Appendix 2

Cost-benefit analysis video, Part 1.

## Multimedia Appendix 3

Cost-benefit analysis video, Part 2.

## Multimedia Appendix 4

Cost-benefit analysis video, Part 3.

## Multimedia Appendix 5

Cost-benefit analysis video, Part 4.

## Multimedia Appendix 6

The ABC exercise video.

## Multimedia Appendix 7

CONSORT-EHEALTH checklist V1.6.2 [40].

## Abbreviations

ANCOVA: analysis of covariance
AUDIT: Alcohol User Disorders Identification Test
BSI: Brief Symptom Inventory
DDD: mean standard drinks per drinking day
DrInC: Drinker’s Inventory of Consequences
DWI: driving while intoxicated
InDUC: Inventory of Drug Use Consequences
OA: Overcoming Addictions Web application
PDA: percent days abstinent (in previous 90 days)
SMART: Self-Management And Recovery Training
SO: significant other
SR: SMART Recovery meetings
TLFB: Timeline Follow-Back
