# Supplementary material for: Overcoming Addictions, a Web-Based Application, and SMART Recovery, an Online and In-Person Mutual Help Group for Problem Drinkers, Part 1: Three-Month Outcomes of a Randomized Controlled Trial
Source: J Med Internet Res. 2013 Jul 11;15(7):e134. doi: 10.2196/jmir.2565 (PMC3713880; doi:10.2196/jmir.2565)
Supplement: Supplementary file 7 [file jmir_v15i7e134_app7.pdf]

CONSORT-EHEALTH Checklist V1.6.2 Report

(based on CONSORT-EHEALTH V1.6), available at [http://tinyurl.com/consort-ehealth-v1-6].

2565

Date completed
2/4/2013 15:24:28
by
Reid Hester

Overcoming Addictions, a web-based application & SMART Recovery, an online and in-person mutual help group: Three month outcomes of a randomized clinical trial

TITLE

1a-i) Identify the mode of delivery in the title
"Overcoming Addictions, a web-based application & SMART Recovery, an online and in-person mutual help group: Three month outcomes of a randomized clinical trial"

1a-ii) Non-web-based components or important co-interventions in title
"Overcoming Addictions, a web-based application & SMART Recovery, an online and in-person mutual help group: Three month outcomes of a randomized clinical trial"

1a-iii) Primary condition or target group in the title
"Overcoming Addictions, a web-based application & SMART Recovery, an online and in-person mutual help group: Three month outcomes of a randomized clinical trial"

The word Addictions in the title reflects the target issue

ABSTRACT

1b-i) Key features/functionalities/components of the intervention and comparator in the METHODS section of the ABSTRACT
"We recruited 189 heavy problem drinkers primarily through SMART Recovery's web site and in-person meetings throughout the U.S. We randomly assigned participants to: OA alone; OA+ attend SMART Recovery meetings (OA +SR), or to attend SMART Recovery meetings (SR) only. Baseline and follow-ups were conducted via GoToMeeting sessions with a Research Assistant (RA) and the study participant. We interviewed significant others to corroborate the participant's self-report. Primary outcome measures include percent days abstinent (PDA), mean drinks per drinking day (DDD), and alcohol/drug related consequences. "

Blinding status was not mentioned in the abstract in light of the length restriction but described in the Methods section of the paper itself.

1b-ii) Level of human involvement in the METHODS section of the ABSTRACT
"We randomly assigned participants to: OA alone; OA+ attend SMART Recovery meetings (OA +SR), or to attend SMART Recovery meetings (SR) only. Baseline and follow-ups were conducted via GoToMeeting sessions with a Research Assistant (RA) and the study participant."

We did not mention the level of training of meeting facilitators in the abstract in light of its length restriction.

1b-iii) Open vs. closed, web-based (self-assessment) vs. face-to-face assessments in the METHODS section of the ABSTRACT
"Baseline and follow-ups were conducted via GoToMeeting sessions with a Research Assistant (RA) and the study participant."

1b-iv) RESULTS section in abstract must contain use data
"We recruited 189 heavy problem drinkers primarily through SMART Recovery's web site and in-person meetings throughout the U.S.....

Actual use relationships were found, for the OA groups, between SR online meetings and improvement in PDA ($r = .261$, $P = .033$). In addition, in the OA groups, the number of total sessions of support (incl. SR & other meetings, counselor visits) was significantly related to PDA ($r = .306$, $P = .012$) and amount of improvement in alcohol-related problems ($r = .305$, $P = .012$). In the SR only group, the number of face to face meetings was significantly related to all three dependent variables, and predicted increased PDA ($r =.358$, $P=.003$), fewer mean DDD ($r =-.250$, $P = .039$) and fewer alcohol-related problems ($r = -.244$, $P= .045$), as well as to the amount of improvement in all three of these variables."

1b-v) CONCLUSIONS/DISCUSSION in abstract for negative trials
"These results support our first experimental hypothesis but not the second. All groups significantly increased their PDA and decreased both their mean DDD and their alcohol-related problems. This suggests that both interventions being investigated were effective in helping people recover from their problem drinking."

INTRODUCTION

2a-i) Problem and the type of system/solution
"Individuals with Addictions in Search of Alternatives. While the predominant model for addressing addictive behaviors in the U.S. is the 12-step model (e.g., Alcoholics Anonymous, Narcotics Anonymous, etc.), a significant proportion of individuals who are looking for help with their addictions reject 12-step programs for a variety of reasons (Institute of Medicine, 1990). At least some of these individuals are interested in viable alternative recovery options--often preferring approaches that provide them with more flexibility in how they define and address their addictive behavior(s). SMART Recovery (Self-Management And Recovery Training [2] provides such individuals with a protocol that, like a 12-step program, employs the use of an interactive group component (either in person or through the use of web-based "chat" rooms and a forum) while using the framework of the 4 point program (described below). However, SMART Recovery fundamentally differs from the 12-step model in that it: 1) encourages a science-based approach to recovery; 2) avoids labeling (e.g., "alcoholic" or addict" unless individuals themselves accept that label); and 3) does not conceptualize addiction as a disease, per se (but is accepting of members' views of addiction as a disease). Anecdotal evidence from SMART Recovery meetings indicates that these aspects of the program draw participants to SMART Recovery (A.T. Horvath, personal communication, 12/2/08).

The Overcoming Addictions (OA) web application. OA is an abstinence-focused, cognitive-behavioral web application we designed for SMART Recovery. The program has parallel but separate modules for alcohol, marijuana, opioids, stimulants, and compulsive gambling. The interactive exercises in OA include tasks that focus on the 4-point program of SMART Recovery as well as additional activities to: enhance motivation for change; track urges over time (with feedback); practice mindfulness exercises for preventing relapse (Bowen et al., 2006), set goals, and make Change Plans (Miller, Zweben, DiClemente & Rychtarik, 1995)."

2a-ii) Scientific background, rationale: What is known about the (type of) system
"While the predominant model for addressing addictive behaviors in the U.S. is the 12-step model (e.g., Alcoholics Anonymous, Narcotics Anonymous, etc.), a significant proportion of individuals who are looking for help with their addictions reject 12-step programs for a variety of reasons (Institute of Medicine, 1990). At least some of these individuals are interested in viable alternative recovery options--often preferring approaches that provide them with more flexibility in how they define and address their addictive behavior(s). "

METHODS

3a) CONSORT: Description of trial design (such as parallel, factorial) including allocation ratio

"To evaluate the effectiveness of OA and SMART Recovery, we conducted a randomized clinical trial."

Hypotheses from Abstract
"Our experimental hypotheses are: 1) All groups will reduce their drinking and alcohol/drug related consequences at follow-up compared to their baseline levels; 2) the experimental groups (OA, OA +SR) will reduce their drinking and alcohol/drug related consequences more than the control group (SR)."

3b) CONSORT: Important changes to methods after trial commencement (such as eligibility criteria), with reasons

"After the first three months though, we stopped randomizing participants to the OA only group and we started encouraging those who had been assigned to this group to attend SR meetings. We did this because recruitment was slow and feedback from referral sources at SMART Recovery indicated that many potential participants were unwilling to be randomized to a condition that asked them to not attend SR meetings."

3b-i) Bug fixes, Downtimes, Content Changes

4a) CONSORT: Eligibility criteria for participants

It seems that the answers to this section are described in the subsections.

4a-i) Computer / Internet literacy

i "have a computer at home with Internet access" was the only inclusion criteria related to internet literacy"

4a-ii) Open vs. closed, web-based vs. face-to-face assessments:

ii "Participants were recruited through a home page announcement on SMART Recovery's web site, announcements of the study at SMART Recovery face-to-face and on-line group meetings nationally, and on their blog. We also placed a thread on the SMART Recovery online forum announcing the study and invited individuals who were new to SMART Recovery to participate in the study...

Potential participants were screened over the phone using a questionnaire addressing the inclusion criteria 1 and 4-6 and exclusion criteria questions 1-7. The research assistant administered the AUDIT over the phone, and asked two quantity/frequency questions, “How often have you had 5 or more (4 or more for women) standard drinks (explained briefly) in the last 90 days?” and “During the last 90 days have you drank as often as once a month?” A response of one or more times to both questions was sufficient to be included in the study. These two screening questions were adapted from those used by Cherpitel (2002), who found them sensitive and specific in screening for alcohol abuse and dependence. We also included a question regarding suicidal thoughts, intent or behaviors. If a participant endorsed this item, we discussed ways to access support (e.g. National Suicide Hotline). We emailed potential participants a demographic form, a patient locator form, a copy of the Brief Symptom Inventory (BSI, Derogatis, 2000) and an Informed Consent form. BSI scores were reviewed prior to enrolling potential participants in the study; if their scores were elevated and the participant reported significant levels of distress, they were encouraged to access professional support (e.g.ABCT web site). Potential participants who screened positive, had a consenting SO and signed the Informed Consent form were randomized to either the experimental or the control groups...

After participants completed and returned the consent form, BSI, Participant Locator, and Demographics forms, they were scheduled for a baseline interview. We used GoToMeeting.com to complete the interview. This program allows sharing of the interviewer's screen so the assessment can be viewed by both parties. Participants used the TLFB calendar generated to prompt recall of their prior three months of drinking as the RA entered their data in a web application we developed for collecting data for this study, the Drinker's Evaluation. Participants then were guided to the InDuC and asked to complete it. At the completion of the interview, they were randomly assigned to a group. Participants and research staff were not blinded to group allocation.
Participants often wanted to discuss their histories and current struggles during the assessments. In order to limit the effect of the assessment interaction, RAs responded empathically but as briefly as possible, without soliciting further processing by the participant. Further, RAs directed, as indicated, that the participant seek help from the interventions being tested in the trial. All participants received a welcome email to the study. For those assigned to the OA conditions, there was a link to the OA registration page. For those assigned to meetings, a link to the SMARTrecovery.org website was provided to facilitate locating available meetings."

4a-iii) Information giving during recruitment

"Screening. Potential participants were screened over the phone using a questionnaire addressing the inclusion criteria 1 and 4-6 and exclusion criteria questions 1-7. The research assistant administered the AUDIT over the phone, and asked two quantity/frequency questions, “How often have you had 5 or more (4 or more for women) standard drinks (explained briefly) in the last 90 days?” and “During the last 90 days have you drank as often as once a month?” A response of one or more times to both questions was sufficient to be included in the study. These two screening questions were adapted from those used by Cherpitel (2002), who found them sensitive and specific in screening for alcohol abuse and dependence. We also included a question regarding suicidal thoughts, intent or behaviors. If a participant endorsed this item, we discussed ways to access support (e.g. National Suicide Hotline).
We emailed potential participants a demographic form, a patient locator form, a copy of the Brief Symptom Inventory (BSI, Derogatis, 2000) and an Informed Consent form. BSI scores were reviewed prior to enrolling potential participants in the study; if their scores were elevated and the participant reported significant levels of distress, they were encouraged to access professional support (e.g.ABCT web site). Potential participants who screened positive, had a consenting SO and signed the Informed Consent form were randomized to either the experimental or the control groups."

4b) CONSORT: Settings and locations where the data were collected

"Baseline Interview. After participants completed and returned the consent form, BSI, Participant Locator, and Demographics forms, they were scheduled for a baseline interview. We used GoToMeeting.com to complete the interview. This program allows sharing of the interviewer's screen so the assessment can be viewed by both parties. Participants used the TLFB calendar generated to prompt recall of their prior three months of drinking as the RA entered their data in a web application we developed for collecting data for this study, the Drinker's Evaluation. Participants then were guided to the InDuC and asked to complete it. At the completion of the interview, they were randomly assigned to a group. Participants and research staff were not blinded to group allocation.
Participants often wanted to discuss their histories and current struggles during the assessments. In order to limit the effect of the assessment interaction, RAs responded empathically but as briefly as possible, without soliciting further processing by the participant. Further, RAs directed, as indicated, that the participant seek help from the interventions being tested in the trial. All participants received a welcome email to the study. For those assigned to the OA conditions, there was a link to the OA registration page. For those assigned to meetings, a link to the SMARTrecovery.org website was provided to facilitate locating available meetings."

4b-i) Report if outcomes were (self-)assessed through online questionnaires

"Baseline Interview. After participants completed and returned the consent form, BSI, Participant Locator, and Demographics forms, they were scheduled for a baseline interview. We used GoToMeeting.com to complete the interview. This program allows sharing of the interviewer's screen so the assessment can be viewed by both parties. Participants used the TLFB calendar generated to prompt recall of their prior three months of drinking as the RA entered their data in a web application we developed for collecting data for this study, the Drinker's Evaluation. Participants then were guided to the InDuC and asked to complete it. At the completion of the interview, they were randomly assigned to a group. Participants and research staff were not blinded to group allocation.
Participants often wanted to discuss their histories and current struggles during the assessments. In order to limit the effect of the assessment interaction, RAs responded empathically but as briefly as possible, without soliciting further processing by the participant. Further, RAs directed, as indicated, that the participant seek help from the interventions being tested in the trial. All participants received a welcome email to the study. For those assigned to the OA conditions, there was a link to the OA registration page. For those assigned to meetings, a link to the SMARTrecovery.org website was provided to facilitate locating available meetings."

4b-ii) Report how institutional affiliations are displayed

No

5) CONSORT: Describe the interventions for each group with sufficient details to allow replication, including how and when they were actually administered

5-i) Mention names, credential, affiliations of the developers, sponsors, and owners

Only on the title page and in the Conflict of Interest section below.

"The senior author holds the copyright and patent pending to the Overcoming Addictions web application. He is also a member of SMART Recovery's International Board of Advisors, a volunteer position."

5-ii) Describe the history/development process

Space limitations prevented a discussion of this.

5-iii) Revisions and updating

Space limitations prevented a discussion of this.

5-iv) Quality assurance methods

No.

5-v) Ensure replicability by publishing the source code, and/or providing screenshots/screen-capture video, and/or providing flowcharts of the algorithms used

See attached multimedia file.

5-vi) Digital preservation

Site URL is referenced with WebCite.

5-vii) Access

"Participants could access OA anywhere or anytime they had an Internet connection. Reviewers wishing to access the program can contact the senior author for a reviewer's access login."

5-viii) Mode of delivery, features/functionalities/components of the intervention and comparator, and the theoretical framework

"Description of the Intervention: SMART Recovery. SMART Recovery's protocol for change combines motivational enhancement with cognitive-behavioral principles and strategies for behavior change. Its four point program focuses on: 1) building and maintaining motivation; 2) dealing with urges; 3) managing thoughts, feelings, and behaviors; and 4) cultivating a lifestyle balance (of short and long-term rewards) to prevent relapse.
SMART Recovery's program uses a common set of strategies to address all addictive behaviors. Their rationale for this is based on two aspects of addiction: 1) common etiological factors in both the development and maintenance of addictive behaviors (e.g., affect regulation); and 2) the broad applicability of cognitive-behavioral and motivational strategies that are supported by outcome research across addiction treatments. For instance, alcohol, drugs, and compulsive behaviors like gambling produce powerfully reinforcing changes in affective states, at least on a short-term basis. Identifying these immediate positive consequences is an important step in developing more adaptive alternatives.
SMART Recovery's menu of cognitive-behavioral and motivational strategies has been adapted from treatment interventions and addresses the different Stages of Change (Connors et al., 2001). The SMART Recovery protocol, however, is not a direct outgrowth of the Stages of Change model. Rather, it has gradually evolved based on empirical evidence of "what works" in helping people with addictions. Its elements are designed to help members address issues ranging from basic motivation for change to qualitative lifestyle changes intended to reduce the appeal of, and engagement in, harmful addictive behaviors.
SMART Recovery has a large and active online presence. In 2012 their web site had, on average, 69,786 visits per month and 991 new subscribers on their online forum each month. The message boards now have over 50,000 registered users (a 130% increase in the last 2 years). In addition to their online presence, they have over 800 in-person support groups world-wide.
Description of the Intervention: Overcoming Addictions (OA). OA is an action stage app that is designed to help users learn how to achieve and maintain abstinence. It is a self-directed and interactive web application developed to be used either as a stand-alone intervention, an adjunct to attending SMART Recovery meetings, or as an adjunct to professional therapy for addictions. Participants could access OA anywhere or anytime they had an Internet connection. Reviewers wishing to access the program can contact the senior author for a reviewer's access login.
The OA program contains and extends the elements of the 4 point program of SMART Recovery. Prior to registering a user can read an overview of the program and its relationship to SMART Recovery. During registration, users provide a first name, gender, email address which is also their login username, and password. Once registration is completed, the program creates a new record in its database and personalizes content for that user (e.g., Welcome back, John). The user is then taken to a homepage that lists all of the program's exercises and materials that are grouped by focus. The user can access any module of the program in any order that he or she chooses (see Figure 1 for a screen shot of a user's home page).
The first module, Getting Started gives an overview of the program and provides a discussion of the Stages of Change (reference) and suggests exercises based on the individual's stage.
The second module, Building and Maintaining Motivation for Change, contains a values exercise, a decisional balance exercise that asks users to weigh the pros and cons of changing, and a cost-benefit analysis exercise that is designed to elicit "change talk" from the user.
The third module, Dealing with Urges and Cravings, begins with a brief discussion of urges and their relationship to sobriety and lapses/relapses. It teaches users to self-monitor their urges to use, noting the date, time, intensity, and duration of the urge, the trigger to the urge, how they handled the urge, and their reactions to how they handled it. Users are able to print out a page of self-monitoring cards so that they can easily collect these data as urges happen during their day. Later, when users enter their self-monitoring data, they are provided with graphic feedback about the frequency, intensity, and duration of their urges over time. This feedback can help users see whether they're making progress in experiencing fewer urges over time. If a user is not experiencing a gradual decline in the frequency, intensity, or duration of urges over time, the program suggests they consider additional or alternative urge-coping strategies. The module also contains the urge-coping strategies recommended by SMART Recovery, empirically supported mindfulness/relaxation exercises, and a section on medications that can help reduce urges and cravings.
In addition, exercises are available to help users identify and manage the triggers that precede urges. Identifying triggers is similar to the first step in a functional analysis of drinking behaviors (Meyers & Smith, 1995) and users are encouraged to develop plans for managing the triggers they personally identify. It is a complex module since of triggers range from simple (e.g., wanting to drink more with some friends than others) to complex (e.g., negative mood coupled with poor coping skills). For each domain of triggers, the program presents strategies that others have found to be helpful.
The fourth module is Self-Managing Thoughts, Behaviors, and Feelings. There are three exercises in this module: 1) the "ABCs" of Rational Emotive Behavior Therapy (REBT) (Ellis & Velten, 1992); 2) unconditional self-acceptance; and 3) problem solving. The ABCs of REBT section has multiple sub-components: dysfunctional beliefs; coping statements; changing one's self-talk to change one's feelings; and the process of analyzing and correcting dysfunctional beliefs that produce negative affect (Steinberger, 2004).
The fifth module is Lifestyle Balance for Preventing Relapse. This module has five components: regaining one's health; relaxation; goal setting; social and recreational activities; and other relapse prevention strategies. The section on regaining one's health focuses on eating and sleeping well, and exercising. The section on relaxation training targets both those with high levels of trait anxiety as well as those sensitive to situation specific arousal (e.g., when experiencing urges to drink/use). The goal-setting component focuses on setting short-term goals that are specific, measurable, achievable, realistic, and timed (e.g., once a day). The section on social and recreational activities helps individuals consider and sample enjoyable and rewarding pro-social activities that are compatible with their goals and values, and that make a sober life more rewarding than drinking, using drugs, or engaging in other addictive behaviors. The section on relapse prevention strategies presents relapse as a learning experience (e.g., the Abstinence Violation Effect, Marlatt & Gordon, 1985) and offers some additional strategies that have not been covered in the other modules.
The appearance of the site is pleasant and uncluttered. Content is delivered via text, embedded videos and audio files, links to other sites, pop-up windows and graphic feedback charts. The site is structured in the hybrid style, meaning that all content is available from a central matrix homepage. Once users choose a content area, their exploration of the content is constrained by tunnels that direct them through the various exercises. At the conclusion of an exercise, users have the option of continuing on to the next recommended activity, or they may return to the homepage."

5-ix) Describe use parameters

"Like most computer-delivered interventions, users are free to access as much program content, in any order, and whenever they choose. Their use is supported by a customizable text messaging and email system that prompts them to log onto the program, reminds them of their plans for managing triggers, reiterates their reasons for staying sober, or presents motivational thoughts. These personalized messages can be delivered daily at user-defined times. "

5-x) Clarify the level of human involvement

"OA is an action stage app that is designed to help users learn how to achieve and maintain abstinence. It is a self-directed and interactive web application developed to be used either as a stand-alone intervention, an adjunct to attending SMART Recovery meetings, or as an adjunct to professional therapy for addictions. "

5-xi) Report any prompts/reminders used

"Their use is supported by a customizable text messaging and email system that prompts them to log onto the program, reminds them of their plans for managing triggers, reiterates their reasons for staying sober, or presents motivational thoughts. These personalized messages can be delivered daily at user-defined times....

The OA program has an integrated email feature that contacts users who have not logged into the program in a week. A personalized email encourages participants to log in and resume their progress through the program. There was no protocol for encouraging participants to attend their SMART Recovery meetings. "

5-xii) Describe any co-interventions (incl. training/support)

No, there were none.

6a) CONSORT: Completely defined pre-specified primary and secondary outcome measures, including how and when they were assessed

"We used the Timeline Follow-Back (TLFB) (Sobell et al., 1979; Sobell & Sobell, 1995) to measure quantity/frequency of alcohol, drug, and tobacco use. The 90-day TLFB was administered at baseline and again at 3- and 6-month post-baseline, which provided continuous data for a total of 9 months. The TLFB was also used to collect data on study participants' attendance at SMART Recovery meetings and other recovery oriented activities in which they may have engaged. We used the Inventory of Drug Use Consequences (InDUC) to measure both lifetime and recent (last 3 months) alcohol- and drug-related consequences. The psychometric properties are described in the manual for the DrInC that was developed for Project MATCH (Miller, Tonigan, & Longabaugh, 1995). "

6a-i) Online questionnaires: describe if they were validated for online use and apply CHERRIES items to describe how the questionnaires were designed/deployed

No. Assessments were conducted online in collaboration with an RA who took participants through assessments via GoToMeeting and a phone call.

6a-ii) Describe whether and how "use" (including intensity of use/dosage) was defined/measured/monitored

"Because study participants could use these resources as much or as little as they chose to, we examined changes over time and treatment group effects for those actually using the resources of the assigned treatment, and examined relationships between engagement (e.g., logging into OA, attending SR and other meetings, and counselor visits) and outcomes."

6a-iii) Describe whether, how, and when qualitative feedback from participants was obtained

Qualitative data from users is only being collected at the 6 month follow-up and will be reported then.

6b) CONSORT: Any changes to trial outcomes after the trial commenced, with reasons

n/a

7a) CONSORT: How sample size was determined

7a-i) Describe whether and how expected attrition was taken into account when calculating the sample size

No. The suggested guidelines for word limits prevented us from presenting our power analysis.

7b) CONSORT: When applicable, explanation of any interim analyses and stopping guidelines

No.

8a) CONSORT: Method used to generate the random allocation sequence

We used a computer generated stratification process for randomization. Participants were classified into blocks based on gender and ethnicity (White, Hispanic, or Other). Within each block, participants were randomly assigned to one of the three groups."

8b) CONSORT: Type of randomisation; details of any restriction (such as blocking and block size)

See above. No restrictions.

9) CONSORT: Mechanism used to implement the random allocation sequence (such as sequentially numbered containers), describing any steps taken to conceal the sequence until interventions were assigned

"Participants and research staff were not blinded to group allocation."

10) CONSORT: Who generated the random allocation sequence, who enrolled participants, and who assigned participants to interventions

"At the completion of the interview, they were randomly assigned to a group. " Previous to this comment, the Research Ass't interview had been described.

11a) CONSORT: Blinding - If done, who was blinded after assignment to interventions (for example, participants, care providers, those assessing outcomes) and how

11a-i) Specify who was blinded, and who wasn't

"Participants and research staff were not blinded to group allocation."

11a-ii) Discuss e.g., whether participants knew which intervention was the "intervention of interest" and which one was the "comparator"

The Informed Consent indicated that we were interested in studying the effectiveness of both interventions.

11b) CONSORT: If relevant, description of the similarity of interventions

"The OA program contains and extends the elements of the 4 point program of SMART Recovery."

12a) CONSORT: Statistical methods used to compare groups for primary and secondary outcomes

"We did not use imputation to account for missing data. Consistent with intent to treat analyses, we examined the entire sample as well as examined changes within the randomly assigned groups. In addition, we formed groups based on their use of either SMART Recovery meetings or the OA application to examine actual use outcomes."

12a-i) Imputation techniques to deal with attrition / missing values

"We did not use imputation to account for missing data. Consistent with intent to treat analyses, we examined the entire sample as well as examined changes within the randomly assigned groups. In addition, we formed groups based on their use of either SMART Recovery meetings or the OA application to examine actual use outcomes."

12b) CONSORT: Methods for additional analyses, such as subgroup analyses and adjusted analyses

"In addition, we formed groups based on their use of either SMART Recovery meetings or the OA application to examine actual use outcomes."

RESULTS

13a) CONSORT: For each group, the numbers of participants who were randomly assigned, received intended treatment, and were analysed for the primary outcome

See flowchart of participant flow and follow-up figure 2

13b) CONSORT: For each group, losses and exclusions after randomisation, together with reasons

See flowchart of participant flow and follow-up figure 2

13b-i) Attrition diagram

See flowchart of participant flow and follow-up figure 2

14a) CONSORT: Dates defining the periods of recruitment and follow-up

"Recruitment began September 12, 2011 (3 pilot participants were recruited in the first 2 weeks of the study) and ended August 1, 2012. Three month follow ups were completed November 1, 2012. Six month follow ups will be completed by March 1, 2013."

14a-i) Indicate if critical "secular events" fell into the study period

No. There were none.

14b) CONSORT: Why the trial ended or was stopped (early)

no, n/a.

15) CONSORT: A table showing baseline demographic and clinical characteristics for each group

See Table 1

15-i) Report demographics associated with digital divide issues

See Table 1

16a) CONSORT: For each group, number of participants (denominator) included in each analysis and whether the analysis was by original assigned groups

16-i) Report multiple “denominators” and provide definitions

"Of the 189 participants who completed random assignment and baseline interviews, 151 (83%) completed the 3 month interview. Of the 37 for whom we do not have 3 month follow-up data, 10 withdrew from the study and 27 were lost to follow-up. Of the 151 with 3 month follow-up data, 83 are in the OA and OA+SR groups and 68 are in the SR group."

16-ii) Primary analysis should be intent-to-treat

"Separate repeated measures analyses of variance were conducted to assess for significance of the change over time. Our three outcome measures were Percent Days Abstinent (PDA), Mean Standard Drinks per Drinking Day (DDD), and the InDUC Recent Total score (InDUC). Improvement over all groups from baseline to 3 months was highly significant on all three dependent variables: PDA, $F(1,149) = 160.93$, $P < .001$, with the mean PDA increasing from 44% to 72%; DDD, $F(1,149) = 61.73$, $P < .001$, with the mean decreasing from 8.0 to 4.6; and InDuC, $F(1,149) = 122.28$, $P < .001$, with the overall mean decreasing from 40.8 to 19.5. However, none of the tests of group differences in change over time approached significance, $F <= 1.0$. The within-group effect sizes (Cohen d) are presented in Table 2. Tests of effects of treatment group were carried out both as tests of Group x Time in a repeated measures approach, and as ANCOVAs. None of the tests that would have been indicative of differential treatment effects approached significance."

See also Table 2 Means and within group effect sizes for each outcome variable

17a) CONSORT: For each primary and secondary outcome, results for each group, and the estimated effect size and its precision (such as 95% confidence interval)

See table 2

17a-i) Presentation of process outcomes such as metrics of use and intensity of use

"Although it was unclear what criterion to use to consider a participant treated, 59 (71%) of the 83 OA+SR participants completing the 3-month follow-up had completed 2 or more OA sessions, and 58 (85%) of the 68 SR participants completing the 3-month follow-up had attended 2 or more SR meetings."

17b) CONSORT: For binary outcomes, presentation of both absolute and relative effect sizes is recommended

No.

18) CONSORT: Results of any other analyses performed, including subgroup analyses and adjusted analyses, distinguishing pre-specified from exploratory

See Table 3 and figures 3-5

"Using these definitions of being actually treated, Improvement of treated participants over all groups from baseline to 3 months was highly significant on all three dependent variables: PDA, F (1,115) = 139.71, P < .001, with the mean PDA increasing from 44% to 73%; DDD, F (1,115) = 55.04, P < .001, with the mean decreasing from 8.3 to 4.4; and InDuC, F (1,115) = 93.95, P < .001, with the overall mean decreasing from 39.6 to 18.7. However, none of the tests of group differences in change over time approached significance, P > .10.
Comparisons of those only using OA with other groups. Although we had to abandon our initial design which included a group that would have used only OA without having the option of participating in any SR meetings, there were 29 of the 83 participants in the OA conditions who did not take part in SR meetings. This allowed post hoc comparisons to be made among three groups: those using only the OA app (n = 29), those who both used the OA app and attended SR meetings (n = 54), and those randomly assigned to SR Only. These three groups did not differ significantly in composition by gender, ethnicity, age, or education. Although there were no significant differences in mean baseline values on our three primary dependent variables, the trend in each case was for those in the OA only group to be more impaired initially than those who attended SR meetings. Repeated measures ANOVAs again indicated highly significant changes over time on all three dependent variables (P < .001), but, more importantly, tests of the group x time interaction were non-significant. As suggested by the plots of means in Figure 3-5, the test for differential change across the three groups did not approach significance for DDD, F (1, 141) = 0.09, P =.919, or for InDuC, F (1, 141) = 0.34, P =.713. For PDA, while the omnibus test of the group x time interaction was non-significant, F (1, 141) = 2.04, P =.134, the plot of means revealed more separation of the groups. In fact the main effect of groups on PDA was significant, F(2, 141) = 3.10, P =.048, because the overall mean PDA in the OA+SR group (63.4) was greater than the average of the other two groups (53.5), F (1, 141) = 4.65, P =.033. However, this resulted in part from the higher mean PDA at baseline in the OA+SR group, because there was not significant evidence of differential improvement across groups. That is, tests of interaction contrasts indicated that not only was the improvement in the SR only group (27.6) not different from that in the OA only group (23.2), F (1, 141) = 0.51, P =.475, but the improvement in the OA+SR group (32.9) was also not significantly larger than the average improvement of the other two groups (25.4), F (1, 141) = 2.41, P =.122

SR meetings or other support. Was the number of SR meetings, other meetings, and counselor visits predictive of the 3-month outcomes or of the improvement from baseline to 3 months for participants in the two groups? There was evidence of this, with the evidence being stronger in the SR only group than in the OA+SR condition.
Although the trend was for the SR Only group to have more days of Face-to-face meetings (3.31), more days of SR Online meetings (5.90), and more days of Any Support (14.85) than the combined OA group (1.82, 4.42, and 12.80, respectively), these were not significantly different across conditions. For the SR only condition, the number of days of Face-to-face meetings reported at 3 months was significantly predictive of all 6 of these outcome measures: PDA at 3 months (r = .358, P = .003), Mean DDD (r = -.250, P = .039) and InDuC Recent Total at 3 months (r = -.244, P = .045), as well as improvement in PDA (r = .274, P = .024), mean DDD (r = .478, P < .001), and improvement in InDuC Recent Total (r = .403, P = .001). On the other hand, for this group, number of days of SR Online meetings was positively related to mean Drinks per Drinking Day at 3 months (r = .260, P = .032). Number of days of any support for the SR group was positively related to PDA at 3 months (r = .260, P = .032) as well as to improvement in PDA (r = .304, P = .012).
In the OA + SR group (i.e., excluding the 16 participants assigned to the OA only condition), neither days of Face-to-face meetings nor days of SR online meetings was significantly related to any of the outcomes at 3 months or to improvement in those variables from baseline. The variable most predictive of outcomes for this group was the number of days of any support, which was significantly related to PDA at 3 months (r = .306, P = .012) and to improvement in InDuC Recent Total (r = .305, P = .012). In addition, number of days of SR OnLine meetings was predictive of improvement in PDA (r = .261, P = .033). Relevant to the anomalous finding of the positive correlation in the SR group between SR Online meetings and mean Drinks per Drinking Day, the correlation between these variables in the OA + SR group was slightly negative using all 67 subjects (r = -.055). However, if the one subject in this group who reported 83 days of online SR meetings were excluded, the correlation between number of SR meeting days and mean Drinks per Drinking Day would have been positive in this group as well (r = .112, P = .372).
Number of OA sessions. The OA sessions completed variable was available only for those participants in the OA conditions. Participants logged into the OA program, on average, 7.2 times (SD = 6.4). To assess whether there was evidence for an engagement-response relationship the number of sessions completed in the first 90 days was correlated with the values of the primary outcome variables at 3 months and with the improvement in those variables from baseline to 3 months. As shown in Table 3 below, none of these six correlations was significant. Number of Days of SR Online meetings was significantly predictive of improvement in PDA for the OA participants (r = .246, P = .025). Further, number of days of any support was significantly correlated with PDA at 3 months (r = .298, P = .006), and with improvement in InDuC Recent Total (r = .220, P = .045)."

18-i) Subgroup analysis of comparing only users

See answer to 18 above.

19) CONSORT: All important harms or unintended effects in each group

No, none found.

19-i) Include privacy breaches, technical problems

No, n/a

19-ii) Include qualitative feedback from participants or observations from staff/researchers

No but we will present qualitiative data along with the 6 month outcomes in a subsequent paper.

DISCUSSION

20) CONSORT: Trial limitations, addressing sources of potential bias, imprecision, multiplicity of analyses

20-i) Typical limitations in ehealth trials

"There are a number of limitations to this study. First, we did not have a no-intervention control group. While we found it neither practically nor ethically feasible to include such a group in our study, the lack of such a comparison group prevents us from being assured that the treatment assigned was the cause of the improvement. Second, we could not separate out the effects of assessment reactivity that, based on participants' anecdotal reports, did sometimes occur as a function of the baseline evaluation. Third, study participants had, on average, a high level of education (mean 16 years). While this seems to be consistent with the heavy drinkers who affiliate SMART Recovery, it potentially limits the generalizability of the outcomes in populations with lower levels of education. Fourth, the requirement for an SO to corroborate the participant's self-report of drinking may have further limited the sample. We considered that requirement necessary though as we had no other way to confirm participants' self-reports of their drinking."

21) CONSORT: Generalisability (external validity, applicability) of the trial findings

21-i) Generalizability to other populations

"Third, study participants had, on average, a high level of education (mean 16 years). While this seems to be consistent with the heavy drinkers who affiliate SMART Recovery, it potentially limits the generalizability of the outcomes in populations with lower levels of education. Fourth, the requirement for an SO to corroborate the participant's self-report of drinking may have further limited the sample. We considered that requirement necessary though as we had no other way to confirm participants' self-reports of their drinking."

21-ii) Discuss if there were elements in the RCT that would be different in a routine application setting

No.
22) CONSORT: Interpretation consistent with results, balancing benefits and harms, and considering other relevant evidence
22-i) Restate study questions and summarize the answers suggested by the data, starting with primary outcomes and process outcomes (use)
"The experimental hypotheses were: 1) All groups will reduce their drinking and alcohol/drug related consequences at follow-up compared to their baseline levels; 2) the experimental groups (OA, OA +SR) will reduce their drinking and alcohol/drug related consequences more than the control group (SR). These results support our first experimental hypothesis but not the second."
22-ii) Highlight unanswered new questions, suggest future research
No.

Other information

23) CONSORT: Registration number and name of trial registry
"Clinical trials.gov ID: NCT01389297"
24) CONSORT: Where the full trial protocol can be accessed, if available
No
25) CONSORT: Sources of funding and other support (such as supply of drugs), role of funders
"Research was supported by a SBIR grant 1R44AA016237 from NIAAA."
X26-i) Comment on ethics committee approval
"This study was approved by the Presbyterian Healthcare Services Institutional Review Board. Consent was obtained by emailing consent forms and asking for participant signature and witness signature. The consent outlined the nature and extent of participation in the trial. Participants were reminded their participation was voluntary, and they could withdraw from the study at any time. In addition, participants would not be identified to anyone outside of the study staff at any time for any reason."
x26-ii) Outline informed consent procedures
"We emailed potential participants a demographic form, a patient locator form, a copy of the Brief Symptom Inventory (BSI, Derogatis, 2000) and an Informed Consent form....

Participants returned the consent forms via email or scanning the documents in and emailing them."
X26-iii) Safety and security procedures
"In addition, participants would not be identified to anyone outside of the study staff at any time for any reason."
X27-i) State the relation of the study team towards the system being evaluated
"The senior author holds the copyright and patent pending to the Overcoming Addictions web application. He is also a member of SMART Recovery's International Board of Advisors, a volunteer position."
